# Bis[3-(dihydroxy­boryl)anilinium] sulfate

**DOI:** 10.1107/S1600536810012092

**Published:** 2010-04-10

**Authors:** Araceli Vega, Rolando Luna, Hugo Tlahuext, Herbert Höpfl

**Affiliations:** a Centro de Investigaciones Químicas, Universidad Autónoma del Estado de Morelos, Av. Universidad 1001, CP 62209, Cuernavaca, Mexico

## Abstract

In the title compound, 2C_6_H_9_BNO_2_^+^·SO_4_^2−^, the dihydroxy­boryl group of one of the two independent boronic acid mol­ecules participates in (B)O—H⋯O_B_ and N—H⋯O_B_ hydrogen bonds, while the second is involved mainly in the formation of the charge-assisted heterodimeric synthon –B(OH)_2_⋯^−^O_2_SO_2_^−^. These aggregates are further connected through N—H⋯O_sulfate_ inter­actions, forming a complex three-dimensional hydrogen-bonded network.

## Related literature

For related salts, see: Braga *et al.* (2003 ▶); Kara *et al.* (2006 ▶); Rogowska *et al.* (2006 ▶); Melendez *et al.* (1996 ▶); Plaut *et al.* (2000 ▶); SeethaLekshmi *et al.* (2006 ▶). For the use of boronic acids in crystal engineering, see: Aakeröy *et al.* (2005 ▶); Filthaus *et al.* (2008 ▶); Fournier *et al.* (2003 ▶); Pedireddi *et al.* (2004 ▶); Rodríguez-Cuamatzi *et al.* (2004*a* ▶,*b* ▶, 2005 ▶, 2009 ▶); Shimpi *et al.* (2007 ▶); Zhang *et al.* (2007 ▶). For a description of the Cambridge Structural Database, see: Allen (2002 ▶). For hydrogen-bond motifs, see: Bernstein *et al.* (1995 ▶).
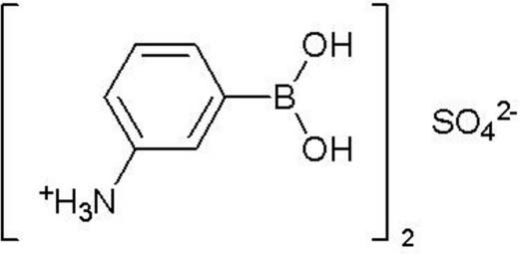


## Experimental

### 

#### Crystal data

2C_6_H_9_BNO_2_^+^·SO_4_^2−^
*M* *_r_* = 371.96
Monoclinic, 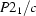

*a* = 5.3589 (9) Å
*b* = 15.695 (3) Å
*c* = 20.489 (3) Å
β = 101.423 (3)°
*V* = 1689.1 (5) Å^3^
*Z* = 4
Mo *K*α radiation
μ = 0.24 mm^−1^
*T* = 173 K
0.41 × 0.18 × 0.09 mm

#### Data collection

Bruker SMART APEX CCD area-detector diffractometer
Absorption correction: multi-scan (*SADABS*; Sheldrick, 1996 ▶) *T*_min_ = 0.83, *T*_max_ = 1.00
18634 measured reflections
3675 independent reflections
2642 reflections with *I* > 2σ(*I*)
*R*_int_ = 0.096

#### Refinement

*R*[*F* ^2^ > 2σ(*F* ^2^)] = 0.078
*wR*(*F* ^2^) = 0.145
*S* = 1.12
3675 reflections
256 parameters
10 restraints
H atoms treated by a mixture of independent and constrained refinement
Δρ_max_ = 0.36 e Å^−3^
Δρ_min_ = −0.37 e Å^−3^

### 

Data collection: *SMART* (Bruker, 2000 ▶); cell refinement: *SAINT-Plus NT* (Bruker, 2001 ▶); data reduction: *SAINT-Plus NT*; program(s) used to solve structure: *SHELXTL-NT* (Sheldrick, 2008 ▶); program(s) used to refine structure: *SHELXTL-NT*; molecular graphics: *SHELXTL-NT*; software used to prepare material for publication: *PLATON* (Spek, 2009 ▶) and *publCIF* (Westrip, 2010 ▶).

## Supplementary Material

Crystal structure: contains datablocks I, global. DOI: 10.1107/S1600536810012092/tk2649sup1.cif

Structure factors: contains datablocks I. DOI: 10.1107/S1600536810012092/tk2649Isup2.hkl

Additional supplementary materials:  crystallographic information; 3D view; checkCIF report

## Figures and Tables

**Table 1:** Hydrogen-bond geometry (Å, °)

*D*—H⋯*A*	*D*—H	H⋯*A*	*D*⋯*A*	*D*—H⋯*A*
O31—H31′⋯O53^i^	0.84 (3)	1.81 (3)	2.653 (4)	175 (3)
O32—H32′⋯O54^i^	0.84 (3)	1.96 (3)	2.744 (4)	155 (3)
N1—H1*A*⋯O54^ii^	0.87 (4)	2.31 (4)	3.161 (5)	169 (4)
N1—H1*C*⋯O52^iii^	0.86 (2)	1.86 (2)	2.718 (4)	176 (5)
O1—H1′⋯O31^iv^	0.84 (4)	1.99 (4)	2.803 (4)	163 (4)
N31—H31*A*⋯O52	0.86 (3)	1.92 (3)	2.787 (4)	179 (3)
N31—H31*C*⋯O2	0.86 (2)	2.07 (2)	2.874 (4)	156 (3)
O2—H2′⋯O32^v^	0.84 (1)	1.99 (2)	2.820 (3)	169 (4)
N1—H1*B*⋯O51^vi^	0.86 (4)	2.13 (4)	2.923 (5)	152 (4)
N1—H1*B*⋯O54^vi^	0.86 (4)	2.37 (4)	3.112 (5)	144 (4)
N31—H31*B*⋯O53^vii^	0.86 (3)	1.90 (3)	2.744 (4)	168 (4)

Symmetry codes: (i) 
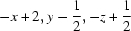
; (ii) 
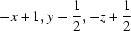
; (iii) 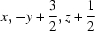; (iv) 
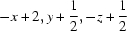
; (v) 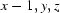; (vi) 
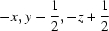
; (vii) 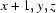.

**Fig. 1.**
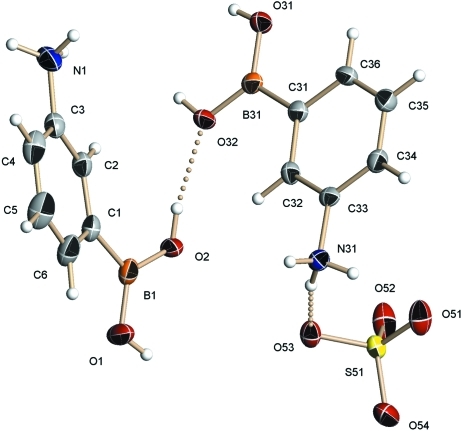
Perspective view of the asymmetric unit of the title compound. Displacement ellipsoids are drawn at the 50% probability level and H atoms are shown as small spheres of arbitrary radii.

**Fig. 2.**
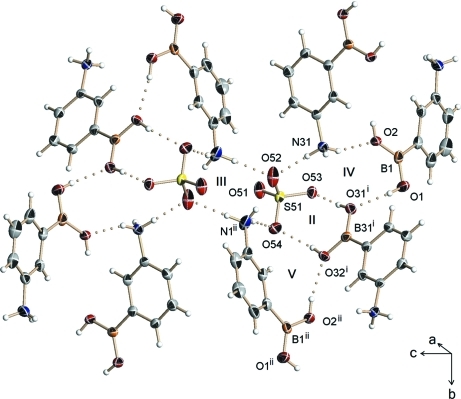
Fragment of the 2D hydrogen-bonded layer in the crystal structure of the title compound, showing motifs II-V. Displacement ellipsoids are drawn at the 50% probability level and H atoms are shown as small spheres ofarbitrary radii. Symmetry operators: (i) -x, 1/2 + y, 1/2 - z; (ii) 1 - x, 1/2 + y, 1/2 - z.

## supplementary crystallographic information

### Comment

Boronic acids, RB(OH)_2_, are capable of forming strong hydrogen bonds with different functional groups such as carboxylic acid and pyridine derivatives (Aakeröy *et al.*, 2005; Pedireddi *et al.*, 2004; Rodríguez-Cuamatzi *et al.*, 2009) and have been employed not only for the formation of neutral homo- and heterodimeric synthons, e.g. RB(OH)_2_···(HO)_2_BR and RB(OH)_2_···HOOCR (Filthaus *et al.*, 2008; Fournier *et al.*, 2003; Rodríguez-Cuamatzi *et al.*, 2004*a*,*b*; Shimpi *et al.*, 2007; Zhang *et al.*, 2007), but also for the generation of charge-assisted synthons such as RB(OH)_2_···^-^OOCR and RB(OH)_2_···^-^OSCR (Kara *et al.*, 2006; Rodríguez-Cuamatzi *et al.*, 2005; Rogowska *et al.*, 2006; SeethaLekshmi *et al.*, 2006).

A search of the CSD (Allen, 2002; version 5.30) revealed that aside from the above-mentioned adducts with organic and inorganic carboxylate derivatives, there are only two further entries for charged motifs of the composition RB(OH)_2_···^-^O_2_E, in which the anions are sulfate and nitrate, respectively (Braga *et al.*, 2003).

The title compound, (I), represents a further example for the –B(OH)_2_···^-^O_2_SO_2_^-^ heterodimeric synthon.

The asymmetric unit of I contains two independent protonated 3-aminophenylboronic acid (3-apba) molecules and one sulfate anion as counterion (Fig. 1). Due to the presence of a large number of hydrogen-bonding functions (BOH, NH_3_^+^ and SO_4_^2-^) a complex 3D hydrogen bonded network is formed, in which the sulfate counterions play the role of the central building block within the crystal structure. Each sulfate is hydrogen bonded to four neighboring [3-apbaH]^+ ^entities through a total of five (*B*)OH···O_sulfate_ and *N*—*H*···O_sulfate_ interactions, and serves as four-connected node (Fig. 2).

Motif II is formed between the –B(OH)_2_ group of one of the two [3-apbaH]^+^ molecules and the sulfate counterion, and corresponds to the charged heterodimeric motif –B(OH)_2_···^-^O_2_SO_2_^- ^[graph set *R*_2_^2^ (8)] (Bernstein *et al.* 1995). In motif III [*R*_4_^4^ (12)] two sulfate groups are hydrogen bridged by two NH_3_^+^ functions. Structurally related hydrogen-bonded rings have been reported previously for secondary ammonium carboxylates (Melendez *et al.*, 1996; Plaut *et al.*, 2000). In motif IV [*R*_4_^4^(12)] three BOH, one sulfate and one NH_3_^+^ group are connected through (*B*)OH···O_sulfate_, (B)OH···O_B_, *N*—*H*···O_sulfate_ and N—H···O_B_ hydrogen bonds , while in motif V [*R*_3_^3^(11)] two BOH, one sulfate and one NH_3_^+^ moiety are connected through (*B*)OH···O_sulfate_, (B)OH···O_B_ and *N*—*H*···O_sulfate_ hydrogen bonds. Motifs II-V give rise to 2D undulated layers (Fig. 2, Table 1), which are connected through three additional *N*—*H*···O_sulfate_ interactions to give an overall 3D hydrogen-bonded network.

### Experimental

The title compound is a commercially available product that has been crystallized from methanol. M.p. > 300 °C.

### Refinement

H atoms were positioned geometrically and constrained using the riding-model approximation [*C*-H_aryl _= 0.93 Å, *U*_iso_(H_aryl_)= 1.2 *U*_eq_(C)]. Hydrogen atoms bonded to O (H1', H2', H31' and H32') and N (H1A, H1B, H1C, H31A, H31B and H31C) were located in difference Fourier maps. The coordinates of the O—H and N—H hydrogen atoms were refined with distance restraints: O—H = 0.840±0.001 Å, N—H = 0.860 ±0.001 Å and [*U*_iso_(H)= 1.5 *U*_eq_(O, N)].

### Crystal data

**Table:**

2C_6_H_9_BNO_2_^+^·SO_4_^2^^−^	*F*(000) = 776
*M**_r_* = 371.96	*D*_x_ = 1.463 Mg m^−^^3^
Monoclinic, *P*2_1_/*c*	Melting point > 573 K
Hall symbol: -P 2ybc	Mo *K*α radiation, λ = 0.71073 Å
*a* = 5.3589 (9) Å	Cell parameters from 1721 reflections
*b* = 15.695 (3) Å	θ = 2.6–20.0°
*c* = 20.489 (3) Å	µ = 0.24 mm^−^^1^
β = 101.423 (3)°	*T* = 173 K
*V* = 1689.1 (5) Å^3^	Rectangular prism, colourless
*Z* = 4	0.41 × 0.18 × 0.09 mm

### Data collection

**Table:**

Bruker SMART APEX CCD area-detector diffractometer	3675 independent reflections
Radiation source: fine-focus sealed tube	2642 reflections with *I* > 2σ(*I*)
graphite	*R*_int_ = 0.096
Detector resolution: 8.3 pixels mm^-1^	θ_max_ = 27.0°, θ_min_ = 1.7°
phi and ω scans	*h* = −6→6
Absorption correction: multi-scan (*SADABS*; Sheldrick, 1996)	*k* = −19→20
*T*_min_ = 0.83, *T*_max_ = 1.00	*l* = −26→26
18634 measured reflections	

### Refinement

**Table:**

Refinement on *F*^2^	Primary atom site location: structure-invariant direct methods
Least-squares matrix: full	Secondary atom site location: difference Fourier map
*R*[*F*^2^ > 2σ(*F*^2^)] = 0.078	Hydrogen site location: inferred from neighbouring sites
*wR*(*F*^2^) = 0.145	H atoms treated by a mixture of independent and constrained refinement
*S* = 1.12	*w* = 1/[σ^2^(*F*_o_^2^) + (0.0357*P*)^2^ + 2.0612*P*] where *P* = (*F*_o_^2^ + 2*F*_c_^2^)/3
3675 reflections	(Δ/σ)_max_ < 0.001
256 parameters	Δρ_max_ = 0.36 e Å^−^^3^
10 restraints	Δρ_min_ = −0.37 e Å^−^^3^

### Special details

**Table:**

Geometry. All esds (except the esd in the dihedral angle between two l.s. planes) are estimated using the full covariance matrix. The cell esds are taken into account individually in the estimation of esds in distances, angles and torsion angles; correlations between esds in cell parameters are only used when they are defined by crystal symmetry. An approximate (isotropic) treatment of cell esds is used for estimating esds involving l.s. planes.
Refinement. Refinement of *F*^2^ against ALL reflections. The weighted *R*-factor wR and goodness of fit *S* are based on *F*^2^, conventional *R*-factors *R* are based on *F*, with *F* set to zero for negative *F*^2^. The threshold expression of *F*^2^ > σ(*F*^2^) is used only for calculating *R*-factors(gt) etc. and is not relevant to the choice of reflections for refinement. *R*-factors based on *F*^2^ are statistically about twice as large as those based on *F*, and *R*- factors based on ALL data will be even larger.

### Fractional atomic coordinates and isotropic or equivalent isotropic displacement parameters (Å^2^)

**Table:**

	*x*	*y*	*z*	*U*_iso_*/*U*_eq_	
B1	0.5330 (8)	0.8900 (3)	0.3774 (2)	0.0287 (10)	
N1	0.3567 (8)	0.6073 (2)	0.47243 (17)	0.0409 (9)	
H1A	0.475 (6)	0.594 (3)	0.451 (2)	0.061*	
H1B	0.230 (6)	0.578 (3)	0.451 (2)	0.061*	
H1C	0.381 (9)	0.588 (3)	0.5125 (8)	0.061*	
O1	0.5531 (6)	0.97477 (16)	0.38584 (13)	0.0415 (8)	
H1'	0.602 (9)	1.000 (3)	0.3546 (16)	0.062*	
O2	0.6184 (5)	0.85326 (15)	0.32467 (12)	0.0271 (6)	
H2'	0.586 (7)	0.8010 (6)	0.320 (2)	0.041*	
C1	0.4023 (7)	0.8355 (2)	0.42509 (17)	0.0277 (9)	
C2	0.4458 (7)	0.7479 (2)	0.43246 (17)	0.0259 (8)	
H2	0.5704	0.7219	0.4119	0.031*	
C3	0.3110 (7)	0.6988 (2)	0.46910 (18)	0.0274 (9)	
C4	0.1327 (7)	0.7344 (3)	0.50092 (19)	0.0350 (10)	
H4	0.0392	0.7000	0.5258	0.042*	
C5	0.0930 (8)	0.8213 (3)	0.4958 (2)	0.0433 (11)	
H5	−0.0273	0.8470	0.5181	0.052*	
C6	0.2248 (8)	0.8711 (3)	0.45906 (19)	0.0379 (10)	
H6	0.1951	0.9308	0.4566	0.045*	
B31	1.3528 (8)	0.6344 (3)	0.2462 (2)	0.0234 (9)	
N31	0.7762 (6)	0.88966 (19)	0.20148 (15)	0.0218 (6)	
H31A	0.664 (5)	0.910 (2)	0.1691 (12)	0.033*	
H31B	0.911 (4)	0.920 (2)	0.2040 (19)	0.033*	
H31C	0.714 (6)	0.894 (2)	0.2370 (10)	0.033*	
O31	1.3885 (5)	0.55072 (15)	0.23406 (12)	0.0259 (6)	
H31'	1.522 (4)	0.530 (2)	0.2572 (17)	0.039*	
O32	1.5200 (5)	0.68048 (15)	0.29159 (12)	0.0272 (6)	
H32'	1.633 (5)	0.649 (2)	0.3138 (17)	0.041*	
C31	1.1043 (7)	0.6786 (2)	0.20739 (16)	0.0221 (8)	
C32	1.0514 (6)	0.7641 (2)	0.21890 (17)	0.0220 (8)	
H32	1.1704	0.7966	0.2495	0.026*	
C33	0.8294 (6)	0.8014 (2)	0.18643 (16)	0.0201 (7)	
C34	0.6537 (7)	0.7561 (2)	0.14105 (17)	0.0254 (8)	
H34	0.5010	0.7825	0.1188	0.030*	
C35	0.7032 (7)	0.6721 (2)	0.12854 (18)	0.0285 (9)	
H35	0.5845	0.6405	0.0971	0.034*	
C36	0.9239 (7)	0.6335 (2)	0.16143 (17)	0.0251 (8)	
H36	0.9541	0.5753	0.1527	0.030*	
S51	0.18611 (16)	0.99469 (6)	0.11838 (4)	0.0218 (2)	
O51	−0.0477 (5)	0.95998 (18)	0.08028 (13)	0.0375 (7)	
O52	0.4070 (5)	0.9546 (2)	0.09827 (13)	0.0452 (8)	
O53	0.2071 (5)	0.97849 (15)	0.19058 (11)	0.0274 (6)	
O54	0.1982 (6)	1.08633 (17)	0.10711 (14)	0.0470 (8)	

### Atomic displacement parameters (Å^2^)

**Table:**

	*U*^11^	*U*^22^	*U*^33^	*U*^12^	*U*^13^	*U*^23^
B1	0.032 (2)	0.033 (3)	0.020 (2)	0.007 (2)	0.0024 (18)	0.0038 (18)
N1	0.066 (3)	0.033 (2)	0.0265 (19)	−0.0244 (19)	0.0168 (19)	−0.0051 (16)
O1	0.077 (2)	0.0217 (15)	0.0305 (16)	0.0007 (14)	0.0210 (15)	0.0008 (12)
O2	0.0406 (16)	0.0190 (13)	0.0232 (13)	−0.0056 (12)	0.0098 (12)	0.0010 (11)
C1	0.031 (2)	0.035 (2)	0.0169 (18)	0.0012 (17)	0.0029 (16)	0.0030 (15)
C2	0.028 (2)	0.032 (2)	0.0184 (18)	−0.0022 (16)	0.0060 (15)	−0.0012 (15)
C3	0.028 (2)	0.033 (2)	0.0207 (19)	−0.0066 (17)	0.0039 (16)	−0.0012 (16)
C4	0.023 (2)	0.057 (3)	0.026 (2)	−0.0051 (19)	0.0057 (17)	0.0067 (19)
C5	0.033 (2)	0.065 (3)	0.034 (2)	0.019 (2)	0.0135 (19)	0.007 (2)
C6	0.041 (2)	0.046 (3)	0.026 (2)	0.014 (2)	0.0045 (18)	0.0082 (19)
B31	0.031 (2)	0.022 (2)	0.020 (2)	0.0009 (18)	0.0101 (18)	0.0015 (17)
N31	0.0212 (16)	0.0228 (16)	0.0224 (16)	0.0006 (13)	0.0065 (13)	0.0024 (13)
O31	0.0284 (15)	0.0223 (14)	0.0254 (14)	0.0043 (11)	0.0013 (11)	−0.0028 (11)
O32	0.0331 (15)	0.0181 (13)	0.0270 (14)	0.0037 (11)	−0.0022 (12)	−0.0040 (11)
C31	0.0243 (19)	0.0234 (19)	0.0192 (17)	−0.0002 (15)	0.0059 (15)	0.0007 (15)
C32	0.0206 (19)	0.026 (2)	0.0186 (18)	−0.0023 (15)	0.0019 (14)	−0.0002 (14)
C33	0.0231 (19)	0.0196 (18)	0.0195 (17)	−0.0019 (14)	0.0091 (15)	0.0025 (14)
C34	0.025 (2)	0.028 (2)	0.0224 (19)	−0.0011 (16)	0.0021 (15)	0.0038 (15)
C35	0.031 (2)	0.027 (2)	0.026 (2)	−0.0054 (17)	0.0014 (17)	0.0007 (16)
C36	0.034 (2)	0.0183 (19)	0.0233 (19)	−0.0013 (16)	0.0067 (16)	−0.0033 (14)
S51	0.0197 (4)	0.0264 (5)	0.0190 (4)	0.0009 (4)	0.0035 (3)	0.0033 (4)
O51	0.0253 (15)	0.0582 (19)	0.0267 (15)	−0.0102 (13)	−0.0002 (12)	0.0024 (13)
O52	0.0317 (16)	0.079 (2)	0.0254 (15)	0.0252 (15)	0.0063 (12)	−0.0004 (14)
O53	0.0279 (14)	0.0339 (15)	0.0205 (13)	−0.0081 (11)	0.0049 (11)	0.0021 (11)
O54	0.071 (2)	0.0271 (16)	0.0368 (17)	−0.0020 (15)	−0.0049 (15)	0.0082 (13)

### Geometric parameters (Å, °)

**Table:**

B1—O1	1.344 (5)	B31—C31	1.571 (5)
B1—O2	1.380 (5)	N31—C33	1.460 (4)
B1—C1	1.565 (6)	N31—H31A	0.86 (3)
N1—C3	1.456 (5)	N31—H31B	0.86 (3)
N1—H1A	0.87 (4)	N31—H31C	0.86 (2)
N1—H1B	0.86 (4)	O31—H31'	0.84 (3)
N1—H1C	0.86 (2)	O32—H32'	0.84 (3)
O1—H1'	0.84 (4)	C31—C32	1.400 (5)
O2—H2'	0.840 (12)	C31—C36	1.401 (5)
C1—C2	1.397 (5)	C32—C33	1.374 (5)
C1—C6	1.401 (5)	C32—H32	0.9500
C2—C3	1.376 (5)	C33—C34	1.381 (5)
C2—H2	0.9500	C34—C35	1.380 (5)
C3—C4	1.378 (5)	C34—H34	0.9500
C4—C5	1.381 (6)	C35—C36	1.380 (5)
C4—H4	0.9500	C35—H35	0.9500
C5—C6	1.374 (6)	C36—H36	0.9500
C5—H5	0.9500	S51—O51	1.445 (3)
C6—H6	0.9500	S51—O54	1.460 (3)
B31—O31	1.358 (5)	S51—O52	1.470 (3)
B31—O32	1.364 (5)	S51—O53	1.483 (2)
			
O1—B1—O2	119.0 (4)	C33—N31—H31A	108 (3)
O1—B1—C1	119.7 (4)	C33—N31—H31B	110 (3)
O2—B1—C1	121.3 (4)	H31A—N31—H31B	107 (3)
C3—N1—H1A	110 (3)	C33—N31—H31C	112 (3)
C3—N1—H1B	113 (3)	H31A—N31—H31C	107 (4)
H1A—N1—H1B	102 (4)	H31B—N31—H31C	111 (4)
C3—N1—H1C	113 (3)	B31—O31—H31'	114 (3)
H1A—N1—H1C	114 (5)	B31—O32—H32'	111 (3)
H1B—N1—H1C	104 (4)	C32—C31—C36	117.5 (3)
B1—O1—H1'	114 (3)	C32—C31—B31	121.2 (3)
B1—O2—H2'	114 (3)	C36—C31—B31	121.3 (3)
C2—C1—C6	117.0 (4)	C33—C32—C31	120.8 (3)
C2—C1—B1	121.3 (3)	C33—C32—H32	119.6
C6—C1—B1	121.6 (4)	C31—C32—H32	119.6
C3—C2—C1	121.0 (4)	C32—C33—C34	121.1 (3)
C3—C2—H2	119.5	C32—C33—N31	119.3 (3)
C1—C2—H2	119.5	C34—C33—N31	119.6 (3)
C2—C3—C4	121.3 (4)	C35—C34—C33	119.1 (3)
C2—C3—N1	118.5 (3)	C35—C34—H34	120.4
C4—C3—N1	120.2 (3)	C33—C34—H34	120.4
C3—C4—C5	118.4 (4)	C36—C35—C34	120.4 (3)
C3—C4—H4	120.8	C36—C35—H35	119.8
C5—C4—H4	120.8	C34—C35—H35	119.8
C6—C5—C4	121.0 (4)	C35—C36—C31	121.1 (3)
C6—C5—H5	119.5	C35—C36—H36	119.4
C4—C5—H5	119.5	C31—C36—H36	119.4
C5—C6—C1	121.3 (4)	O51—S51—O54	110.29 (17)
C5—C6—H6	119.4	O51—S51—O52	110.33 (17)
C1—C6—H6	119.4	O54—S51—O52	108.31 (19)
O31—B31—O32	122.7 (3)	O51—S51—O53	111.12 (15)
O31—B31—C31	118.1 (3)	O54—S51—O53	109.24 (16)
O32—B31—C31	119.2 (3)	O52—S51—O53	107.46 (15)

### Hydrogen-bond geometry (Å, °)

**Table:**

*D*—H···*A*	*D*—H	H···*A*	*D*···*A*	*D*—H···*A*
O31—H31'···O53^i^	0.84 (3)	1.81 (3)	2.653 (4)	175 (3)
O32—H32'···O54^i^	0.84 (3)	1.96 (3)	2.744 (4)	155 (3)
N1—H1A···O54^ii^	0.87 (4)	2.31 (4)	3.161 (5)	169 (4)
N1—H1C···O52^iii^	0.86 (2)	1.86 (2)	2.718 (4)	176 (5)
O1—H1'···O31^iv^	0.84 (4)	1.99 (4)	2.803 (4)	163 (4)
N31—H31A···O52	0.86 (3)	1.92 (3)	2.787 (4)	179 (3)
N31—H31C···O2	0.86 (2)	2.07 (2)	2.874 (4)	156 (3)
O2—H2'···O32^v^	0.84 (1)	1.99 (2)	2.820 (3)	169 (4)
N1—H1B···O51^vi^	0.86 (4)	2.13 (4)	2.923 (5)	152 (4)
N1—H1B···O54^vi^	0.86 (4)	2.37 (4)	3.112 (5)	144 (4)
N31—H31B···O53^vii^	0.86 (3)	1.90 (3)	2.744 (4)	168 (4)

Symmetry codes: (i) −*x*+2, *y*−1/2, −*z*+1/2; (ii) −*x*+1, *y*−1/2, −*z*+1/2; (iii) *x*, −*y*+3/2, *z*+1/2; (iv) −*x*+2, *y*+1/2, −*z*+1/2; (v) *x*−1, *y*, *z*; (vi) −*x*, *y*−1/2, −*z*+1/2; (vii) *x*+1, *y*, *z*.
